# Fish-Based Bioactives as Potent Nutraceuticals: Exploring the Therapeutic Perspective of Sustainable Food from the Sea

**DOI:** 10.3390/md18050265

**Published:** 2020-05-18

**Authors:** Syed Amir Ashraf, Mohd Adnan, Mitesh Patel, Arif Jamal Siddiqui, Manojkumar Sachidanandan, Mejdi Snoussi, Sibte Hadi

**Affiliations:** 1 Department of Clinical Nutrition, College of Applied Medial Sciences, University of Hail, Hail PO Box 2440, Saudi Arabia; amirashrafy2007@gmail.com; 2 Department of Biology, College of Science, University of Hail, Hail PO Box 2440, Saudi Arabia; arifjamal13@gmail.com (A.J.S.); snmejdi@yahoo.fr (M.S.); 3 Bapalal Vaidya Botanical Research Centre, Department of Biosciences, Veer Narmad South Gujarat University, Surat 395007, Gujarat, India; patelmeet15@gmail.com; 4 Department of Oral Radiology, College of Dentistry, University of Hail, Hail PO Box 2440, Saudi Arabia; smanojk68@gmail.com; 5 Laboratory of Bioresources: Integrative Biology and Valorization, (LR14-ES06), University of Monastir, Higher Institute of Biotechnology of Monastir, Avenue Tahar Haddad, BP 74, Monastir 5000, Tunisia; 6 School of Forensic and Applied Sciences, University of Central Lancashire, Preston PR1 2HE, UK

**Keywords:** nutraceutical, functional food, fish, bioactive compounds, omega-3 polyunsaturated fatty acid (PUFA), fatty acids

## Abstract

Recent developments in nutraceuticals and functional foods have confirmed that bioactive components present in our diet play a major therapeutic role against human diseases. Moreover, there is a huge emphasis on food scientists for identifying and producing foods with better bioactive activity, which can ultimately provide wellness and well-being to human health. Among the several well-known foods with bioactive constituents, fish has always been considered important, due to its rich nutritional values and by-product application in food industries. Nutritionists, food scientists, and other scientific communities have been working jointly to uncover new bioactive molecules that could increase the potential and therapeutic benefits of these bioactive components. Despite the innumerable benefits of fish and known fish bioactive molecules, its use by food or pharmaceutical industries is scarce, and even research on fish-based nutraceuticals is not promising. Therefore, this review focuses on the current information/data available regarding fish bioactive components, its application as nutraceuticals for therapeutic purposes in the treatment of chronic diseases, ethnic issues related to consumption of fish or its by-products. Especial emphasis is given on the utilization of fish wastes and its by-products to fulfill the world demand for cheap dietary supplements specifically for underdeveloped/least developed countries.

## 1. Introduction

Traditionally, fish is considered as an affordable source of protein, serving a worldwide population as well as a source of nutraceutical importance [1]. In addition, about one-half of the total biodiversity is comprised of fish and other marine species, which are also a wonderful source for novel bioactive compounds, implicated in the continuous improvement of human health [2]. Several bioactive components present in fish such as lipids, proteins, vitamins, minerals, and other fish by-products are considered important due to their therapeutic potentiality. More importantly, the main therapeutic potential of fish consumption has been ascribed with the presence of long-chain omega-3 polyunsaturated fatty acids (PUFAs) in high content. Several reports suggested that, other than long-chain omega-3 PUFAs, various other bioactive components from fish have been found to have a positive effect on human health [3]. According to the World Health Organization [4] and American Heart Association [5], fish should be consumed on a regular basis (i.e., 1–2 servings weekly). It should provide approximately 200–500 mg dose of eicosapentaenoic acid (EPA) and docosahexaenoic acid (DHA), which can be easily obtained from fish that contain omega-3, for example, salmon or trout. This will protect against various coronary heart diseases and stroke. On the other hand, the nutritional status of fish and fish products shows that they contain high amounts of other healthy nutrients with countless health benefits [6].

Recently, it has been seen that, in developed as well as developing countries, lifestyle-related diseases have become a serious issue to the human population. Various epidemiological data and clinical trials have shown that diet is one of the major factors, other than stress and strain in modern life, influencing lifestyle-related diseases, especially in middle-aged and senile people. Imbalance in lifestyle and diet pattern in developed countries has caused a rise in chronic diseases, such as obesity, diabetes, hypertension, and hyperlipidemia, affecting both wellness and well-being [7]. Therefore, fish is considered as a solution to lifestyle-related diseases. The main objective of this review is to focus on the nutritional significance of fish and fish-based nutraceuticals and functional foods and their therapeutic potential, as well as understand the limitations of fish intake in our dietary habits despite of its myriad of benefits.

## 2. Nutritional Profile of Fish

**Figure 1:**
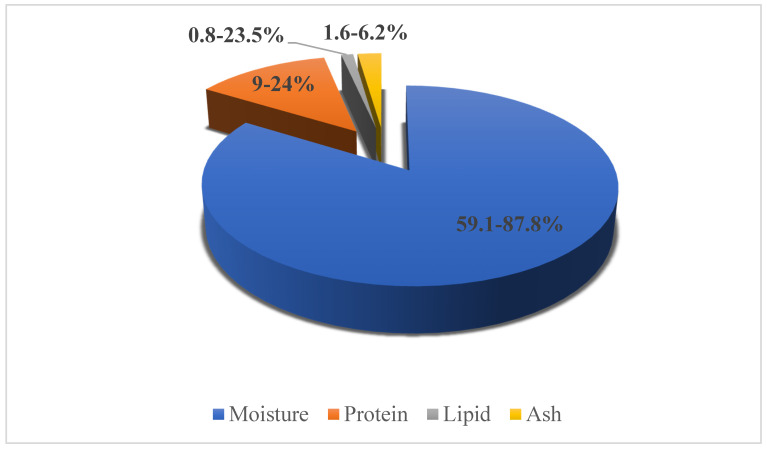
Average mean proximate composition data * from 62 species of fish [13].

**Figure 2:**
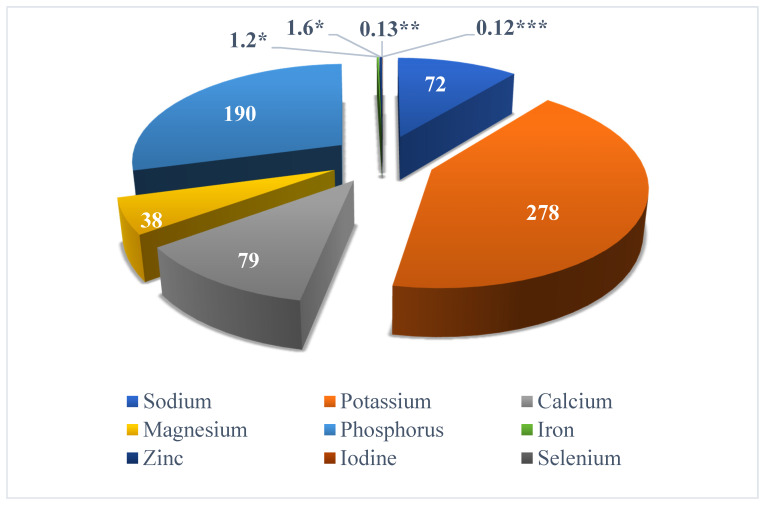
Average mineral composition (mg) of fish [8]. * [11]; ** [14]; *** [15].

Fish is one of the supreme and essential foods in the human diet, due to its eminent nutritional value. Proximate analysis of almost all the fish or fish related foods have been studied by researchers. However, minor discrepancies in nutritional composition exist due to their feeding habits, age, sex, season, temperature, adaptation, and hatching [8]. Further reports and analyses state that fish provide protein of a superior quality with all the essential amino acids, as well as elemental sources of dietary vitamins and minerals, including zinc (marine fish), iron, calcium, phosphorus, iodine, selenium, vitamin A, D, E, several B vitamins (B3, B6, and B12), important amounts of PUFAs and various other micronutrients [9,10,11]. As it is known that most small fishes are eaten whole (with bone, scales, and fins), they are a rich source of abundantly bioavailable calcium. One such example of small fish is *Amblypharyngodon mola*, which is reported to be a rich source of vitamin A, in the form of dihydro-retinol and retinol [12]. Furthermore, bioavailability of zinc and iron from the other foods in a meal is enhanced by fish [10]. The chief chemical composition of fish muscles is protein, water, and lipids, which collectively hold around 98% of the total weight. Meanwhile, average proximate compositions as well as micronutrients of fish are presented in Figure 1 and Figure 2, respectively.

## 3. Fish as Nutraceuticals

**Figure 3:**
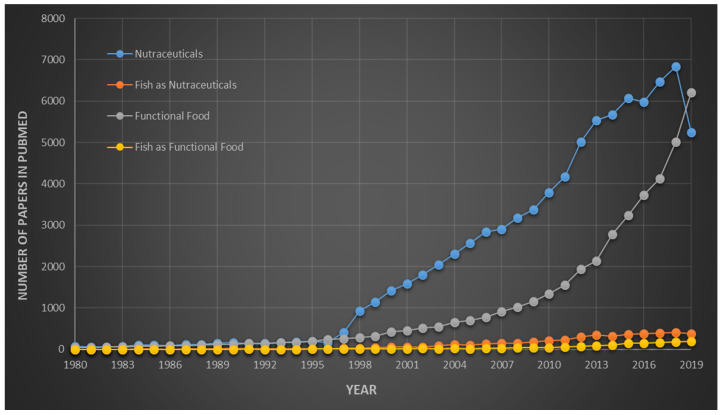
Graphically represented statistical data * of number of publications in PubMed from 1980 to 2019. * Publications in PubMed when using search bar for searching several keywords/phrases: (1) nutraceuticals (blue); (2) fish as nutraceuticals (orange); (3) functional food (grey); and (4) fish as functional food (yellow). Moving average trend lines show the importance and urgent need for research concerning the development of cheap fish/fish wastes/fish by-products-based nutraceuticals and functional foods.

Stephen DeFelice first coined the word “nutraceutical” by combining two words “nutrition” and “pharmaceutical”. He defined nutraceutical as food or a part of food which not only imparts health benefits but also contributes to preventing/treating various diseases [16,17]. Moreover, in broad terms, nutraceuticals play a vital role in human beings by maintaining their normal physiological functions and well-being. Although fish has always been superior and dominant on other protein nutraceuticals and functional foods, research programs on fish-based nutraceuticals and functional foods have reached a saturation point. Even awareness related to its benefits is lacking and should be underpinned [18]. Awareness programs only reach the urban population of countries and somehow the rural population is excluded. Moreover, based on a PubMed search using keywords like nutraceuticals and functional foods, a number of research articles were published at a rapid pace since 1995. However, a change in the search of keywords to fish as nutraceuticals/functional food, the rate of publication has been fairly stagnant since 1980 (Figure 3). This shows the saturation level of this field of study which is a matter of concern. 

Fish nutraceuticals can be used to improve health in various ways: it can delay the ageing process, prevent acute and chronic diseases, increase life expectancy, and support the basic structure and functions of the body [19,20,21]. Urbanization of the population and health awareness among people with either a sedentary lifestyle or stressed lifestyle are the foremost causes of increased growth in the nutraceutical market worldwide [22]. Recent reports suggested that nutraceuticals provide positive tactics to manage healthcare with remarkable beneficial effects on human health [23]. A wide range of phytochemicals such as terpenoids, glucosinolates phytosterols, limonoids, anthocyanidin, polyphenols, phytoestrogens, carotenoids, isoflavonoids, flavonoids, etc., have shown numerous therapeutic effects on human well-being, such as anti-inflammatory, antioxidants, antibacterial, anti-allergic, etc. [24,25,26,27,28,29]. These nutraceuticals alone or in combination with other therapies, not only help in maintaining health and promoting quality of life, but also combat serious medical illnesses of the current era, such as diabetes, cancer, cardiovascular diseases, cholesterol, arthritis, obesity, osteoporosis, etc. [22,30,31]. Therefore, low-cost nutraceuticals have always been in special demand, particularly among economically vulnerable or reduced income groups. This way fish or fish by-products can also solve the global malnutrition problem and related disorders by providing vital micro and macronutrients, high-density fats, and easily digestible proteins [32]. These essential nutrients have many more beneficial physiological roles than other proteins [1].

## 4. Background of Fish Bioactive Compounds and Fishery By-Products

The word "bioactive" is derived from the Greek and Latin words “life” and “dynamic”, full of energy, or involved in an activity [33,34]. Fish, which is an inimitable fusion of bioactive compounds like long-chain PUFAs (EPA and DHA), omega-3 PUFAs, peptides, protein hydrolysates, amino acids, minerals, vitamins, gelatin, collagen, fish oil, fish bone as well as fat-soluble vitamins, makes it an important source of nutraceuticals [34]. Due to the significant therapeutic value of fish and fishery by-products, numerous nutraceuticals are in use for preventive purposes in various fields of medicine. They can be used to lessen complications or to treat various diseases, such as cardiovascular diseases, cancer, viral infections, rickets, dermatologic problems, hypertension, especially during pregnancy, and parasitic infections [35]. Apart from all these benefits, fish-derived nutraceuticals also possess anticoagulant, anti-inflammatory, and antioxidant features [36].

In general, a large amount of fish by-products are either disposed of or used for low-value products [37]. It has been observed that around 25% of fish waste coming from fishery industries are thrown away, which causes a substantial impact on the environment as well as a potential loss of important bioactives present in fish wastes [38]. Fish waste is defined as any fish or fish species with very low or no commercial importance, or fish which are caught in minimal amounts that do not possess much warrant for sale. Moreover, around half of the fish body parts such as head, fins, skin as well as viscera are considered as “waste” in fish industries. According to previous reports, each year the fishery industry generates more than 20 million tons of fish wastes; this is a result of catching non target fishes as well as the fish industry processing waste as well as by-products. These discards represent a huge amount of the total world fish production. In the European Union alone, these fish waste products total 5.2 million tons every year [39,40]. However, three major techniques (chemical or physical, enzymatic method, and microbial method) are employed to recover the bioactive components from these waste products. Among the three techniques, the enzymatic method is considered as one of the best techniques for all types of fish waste (fish head, skin, viscera, frames, and scale) [41]. Consequently, it is important to recognize the value of these fish wastes which can be utilized to develop novel nutraceuticals and functional foods from it. As a result, an increase in research on fish nutraceuticals is required. Cheap valued potent nutraceuticals can fulfill the world demand of dietary supplements, especially for underdeveloped/least developed countries. Table 1 below mentions some nutraceutical components of fish, fish waste, and fish by-products with their health applications.

## 5. Global Fish Consumption and its Nutraceutical Market: Current Scenario and Future Trends

To ensure food security and nutritional quality for a growing world population in the face of climate change, increasing aquaculture production, and competition for natural resources, countries must be accountable for what they consume rather than what they produce [80]. The State of World Fisheries and Aquaculture 2018 highlights the critical importance of fisheries and aquaculture for food and nutrition. The world fish production is 167.2 million tons, out of which 146.3 million tons are used for human consumption and the remaining is used for non-food purposes and discarded as waste material [8,81]. World consumption of fish per capita increased in developing regions from 5.2 kg in 1961 to 18.8 kg in 2013, while in the least developed countries with food deficits it increased from 3.5 kg to 7.6 kg in the same period. Hence, fish accounts for about 17% of the intake of animal protein by the world population [11]. In 2016 global per capita fish consumption rose to above 20 kg a year for the first time, thanks to stronger aquaculture supply and firm demand, record hauls for some key species, and reduced wastage, according to a Food and Agriculture Organization (FAO) report [81].

**Figure 4:**
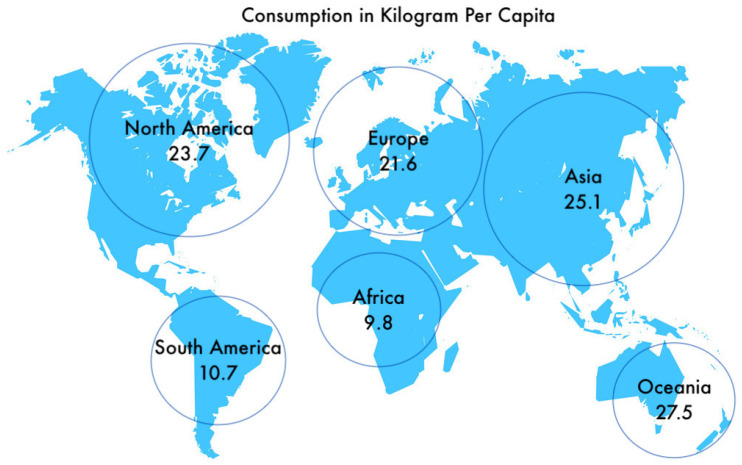
World map showing estimated fish consumption per capita worldwide in 2019 [84].

The global nutraceutical market comprised of functional foods and dietary supplements was valued at around $250 billion in 2014. Consumer demand for nutraceuticals is rapidly increasing with the market expected to reach $722.49 billion by 2027 according to a current report by Grand View Research, Inc. [9,82,83]. Global fish nutraceutical markets comprise mainly of fish oil, as it is the most widely used nutraceutical for human consumption, because of its high content of essential long-chain omega 3 fatty acid. According to the report published by Global Fish Oil Market, the global fish oil market is expected to reach $4.08 billion by 2022 according to a new report by Grand View Research, Inc. [82]. Moreover, other nutraceutical products derived from fishes include collagen, bioactive peptides, protein hydrolysate, carotenoids, and glycosaminoglycans. Figure 4 provides insight about the current fish consumption in kilogram per capita. Africa, with the most least-developed countries in the world, has the smallest consumption rate of fish or fish products. The scientific community and nutraceutical producing global leaders are urged to find a way to use fish wastes and its by-products for developing low-cost nutraceuticals that fulfill almost all the nutritional requirements of a human being. This can be supplied to the least developed nations with properly organized awareness programs by lead organizations like the World Health Organization (WHO).

## 6. Importance and Necessity of Fish Bioactive Components as Dietary Intake

### 6.1. Fatty Acids: Power of Omega 

Fatty acid is a chain of carbon atoms with an acid group (COOH) at one end and an omega or methyl group (CH3) at the other end. They are categorized based on various characteristics, such as length, the existence of double bonds, and the arrangement of hydrogen atoms in double bonds [85]. There are mainly three types of fatty acids: (1) saturated fatty acids (SFAs), (2) monounsaturated fatty acids (MUFAs), and (3) polyunsaturated fatty acids (PUFAs). The SFAs and MUFAs are synthesized endogenously, but PUFAs cannot synthesize by humans from other components by any known biochemical pathways. Therefore, it is must to be obtained from the diet [86]. PUFAs are also called “essential fatty acids” (EFAs), which are given externally through the diet [87]. EFAs help in the formation of healthy cell membranes, proper development and functioning of the brain and nervous system, and production of hormone-like substances called eicosanoid, thromboxane, leukotriene, and prostaglandin. They are also responsible for regulating blood pressure, blood viscosity, vasoconstriction, and immune and inflammatory responses [88,89,90,91,92]. There are two families of PUFAs, and they are classified as omega-3 (n-3) and omega-6 (n-6) based on the location of the last double bond relative to the terminal methyl end of the molecule [93]. Of these, n-3 and n-6 PUFAs play the most important biological roles, and the quantitative balance between n-3 and n-6 PUFAs is believed to be a crucial factor in many disease states including cardiovascular diseases [94].

The major omega-3-fatty acids are α-linolenic acid (ALA), EPA, and DHA. ALA is the precursor of EPA and DHA. EPA and DHA are found mainly in fatty fishes such as mackerel, salmon, herring, trout, blue fin tuna, and in fish oils [22,42]. Omega-3 fatty acids found in fatty fishes are known to be essential in the growth of children and prevention of coronary heart diseases. DHA is important for optimal brain and neurodevelopment in children, while on the other hand, EPA helps in improving cardiovascular health overall. Fatty acids also play an important role in membrane mediated processes such as osmoregulation, nutrient assimilation, and transport [95]. Fish are the most important sources of these fatty acids; fatty fish, such as sardines, mackerel, anchovies, and some salmon species, are rich in EPA and DHA. An important thing to know is that fish cannot synthesize these fatty acids; they obtain them from food they consume (algae and planktons) [96].

**Figure 5:**
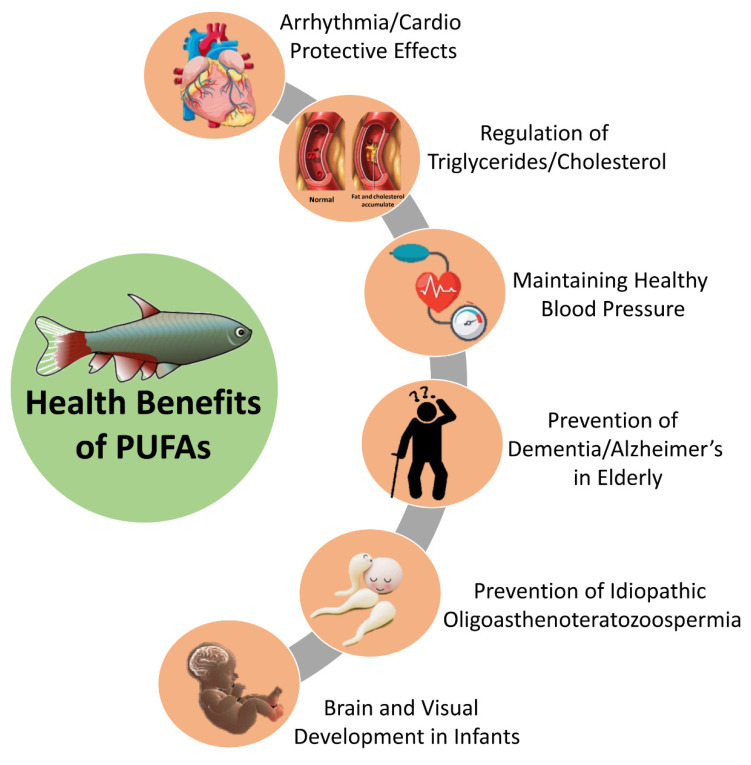
Pictorial representation of health benefits of polyunsaturated fatty acids (PUFAs) in general.

It has been observed that therapeutically important omega-3 PUFAs prevent childhood asthma as well as attention deficit/hyperactivity disorder in children. Moreover, in adults it has been well documented for the prevention of cardiovascular diseases, hypertension, and idiopathic oligoasthenoteratozoospermia. Additionally, in geriatrics, it helps in preventing dementia, age related macular degeneration, Alzheimer’s disease, as well as mood disorder [6,97]. The EPA and DHA fatty acids found in fish also exhibit anti-inflammatory properties through their impact on prostaglandin synthesis [94,98]. For pregnant women, mothers who are breastfeeding, and women of childbearing age, fish intake is highly recommended and important, because it supplies DHA, a specific omega-3 fatty acid that is beneficial for the brain development of infants [99]. Other benefits include anticoagulant effect, help in thinning the blood, as well as antidepressant [100]. The health benefits of omega-3 fatty acids are well-known to scientific, clinical, and industry experts, with research examining effects on almost every body system and for numerous health conditions as mentioned in Table 1 and Figure 5.

### 6.2. Proteins and Amino Acids

Fish is an important source of quality animal proteins (particularly the essential amino acids lysine and methionine). It has been reported that fish protein has a greater satiety effect than other sources of animal proteins like beef and chicken. Around 60% of people from developing countries depend upon fish for over 30% of their animal protein supplies [95,101]. Moreover, fish proteins are highly digestible as well as a rich source of peptides and essential amino acids that are limited in terrestrial meat proteins. Fish proteins have high biological values because of the presence of essential amino acids in good proportions [102]. 

Proteins and amino acids are important biomolecules which regulate key metabolic pathways and serve as precursors for synthesis of biologically important substances [103]. They are also essential for the proper growth and development of the body, including maintenance and repairing of worn out tissues [8]. Most importantly, they play an important role in preventing protein-calorie malnutrition. The protein immunoglobins can act as an important defense against bacterial and viral infections and help in the maintenance of electrolyte and water balance in the human system. The protein derived from fish also balances many body regulatory factors [95]. For example, a sardine protein diet lowered insulin resistance, leptin and tumor necrosis factor (TNF)-α, improved hyperglycemia, and decreased adipose tissue oxidative stress in rats with induced metabolic syndrome. However, due to their undesirable fishy odor and flavor they are mostly used in animal nutrition [3].

Amino acids on the other hand are essential biomolecules that serve as building blocks of proteins as well as intermediates in various metabolic pathways. They are mainly obtained from a protein diet, and the quality of dietary protein is assessed from essential to non-essential amino acid ratio. Deficiency or imbalance in amino acid ratio could affect body metabolism and homeostasis. Thus, by providing adequate amounts of amino acids in the human diet, we can avoid such deficiencies or metabolic disorders [104]. Table 1 shows the list of fish species rich in specific amino acids; deficiency or imbalance of these amino acids leads to various metabolic disorders.

### 6.3. Vitamins, Minerals, and Trace Elements

All the vitamins necessary for human health are present in fish; however, the amount of vitamins may vary depending on fish species. Moreover, a significant source of vitamins A, D, and B is widely present in fish [95]. Vitamin A helps for normal growth, builds cells, prevents the problem of poor eyesight, and helps in the treatment of many eye diseases [105]. Vitamin D in fish is found in the form of vitamin D3 (cholecalciferol) and deficiency of vitamin D could lead to rickets, osteomalacia, and a low bone mineral density (BMD), thereby leading to osteoporosis. Vitamin D deficiency exacerbates osteopenia, osteoporosis, and fractures in adult. In addition to bone related issues, deficiency of vitamin D has been connected with diabetes, increased aggressiveness of certain cancers, and increased occurrence of autoimmune diseases as well as cardiovascular diseases [3].

Abundance of essential mineral and trace elements in fish is due to their ability to obtain inorganic atoms from sea or river water. These essential minerals found in seafood are higher than terrestrial foods [34]. Minerals commonly found in fish flesh are sodium, potassium, calcium, magnesium, phosphorus, sulfur, iron, manganese, zinc, copper, and iodine [106]. Essential elements such as Ca, Mg, Na, K, Fe, Zn, Cu, Mn, Se, F, and iodine, present in human nutrition participate in several biochemical reactions: calcium, magnesium, and phosphorus are crucial in the formation of bones and teeth; sodium and potassium work together in the transmission of nerve impulses and maintaining electrolyte balance; zinc is mostly found as a cofactor in enzyme reactions; and iron forms part of the hemoglobin molecule which transports oxygen around the body [106,107,108].

### 6.4. Carotenoids

Pigments are responsible for the wide spectrum of color in fishes which is an essential prerequisite for the quality, as they fetch a higher price in the commercial market. Carotenoids commonly found in fish are lutein (greenish-yellow), beta carotene (orange), alpha, beta- doradexanthins (yellow), zeaxanthin (yellow-orange), canthaxanthin (orange-red), and astaxanthin (yellow). Among the carotenoids, astaxanthin is very common in red fishes. One such example is the pink coloration of salmon fish, which is due to astaxanthin. It is widely distributed in both marine and freshwater fish. However, lutein pigment is very common in freshwater fish [109]. The importance of the antioxidant properties of carotenoids to human health derives from their potential to reduce the oxidative stress linked to various reactive oxygen species (ROS) related disorders, including various types of cancer, neurological and cardiovascular diseases [64,65]. Another major feature of carotenoids is protection of low-density lipoprotein (LDL) against oxidation, which confers carotenoids’ antiatherogenic properties. Carotenoids (lutein and zeaxanthin) have been shown to prevent eye macula from being damaged by blue light. The beneficial effects of carotenoids have also been shown in patients with psoriasis and skin inflammatory pathology. Carotenoids have been used as preservatives in cosmetics, in combination with other antioxidants or algal bioactive substances, and in creams and lotions for sun protection [110].

### 6.5. Bioactive Peptides and Protein Hydrolysate

Bioactive peptides have been defined as “food derived components, that in addition to their nutritional value exert a physiological effect in the body”. These physiological functions are primarily regulated by some peptides that are encrypted in the native protein sequences [111]. The peptides, which are present in the inactive form within the protein chains, are activated after their hydrolysis using enzymes, including trypsin, proteinases, chymotrypsin, alcalase, and pepsin [112]. The use of bioactive peptides has gained much interest as nutraceuticals and functional foods in recent times. To date, bioactive peptides have shown several therapeutic effects such as antihypertensive, antioxidant, antimicrobials, and antiproliferative effects derived from hydrolysates of meat and fish proteins [66,67]. In addition, other biological activities of bioactive peptides such as antithrombotic, opioid activities, cholesterol-lowering ability, immunomodulating effect, antidiabetic activity, etc., have also been reported by several authors [111,113,114]. Moreover, protein hydrolysates are usually produced by enzymatic hydrolysis of whole protein sources by appropriate proteolytic enzymes under controlled conditions, followed by post-hydrolysis processing to isolate desired and potent bioactive peptides from a complex mixture of active and inactive peptides [111]. Protein hydrolysates and peptides derived from fish by-product waste could be utilized for the prevention and promotion of various chronic diseases [115]. Based on various reports suggesting its potential therapeutic application, these bioactive peptides can exert beneficial health properties and thus are considered as a lead compound for the development of nutraceuticals or functional foods.

### 6.6. Chitin and Chitosan

Chitin is derived from the Greek word ‘chiton’, which means a coat of nail. It is a major exoskeleton component of invertebrates and crustaceans, in which chitin acts as a supportive and protective component. Chitin is the second most plentiful natural polymer on Earth after cellulose. It is distributed in marine invertebrates and is usually extracted from shrimp and crab shell [68,116]. Chitin is chemically composed of N-acetyl D glucosamine and a derivative of glucose. Moreover, chitosan is the simplest and least expensive derivative of chitin, obtained by removing the number of acetyl groups from chitin [117]. Like cellulose, it functions as a structural polysaccharide [118]. The actual variation between chitin and chitosan is the acetyl content of the polymer. Chitin and chitosan have been recognized for their beneficial health effects since the 1980s. Over the past few decades, numerous studies and several clinical trials have been performed which demonstrated the various health benefits: wound healing accelerator, reduce blood cholesterol levels, immune system stimulant, oral therapy-treatment of wounds, anti-ulcer agent, anti-cancer and anti-tumor agent, bactericide, coating agent for prosthetics (artificial parts of the body), antimicrobial activity, antioxidant agent, chitin and chitosan-based dressings, ophthalmology, and anti-ageing cosmetics [68,69,70,119].

In addition, chitin and chitosan are natural, non-toxic, biodegradable polymers with a wide range of biomedical as well as nutraceutical applications. The applicability of chitin and chitosan to a wide range of nutraceutical applications depends on a few factors such as molecular weight and degree of N-acetylation. Moreover, its applicability can also be augmented by promoting the utilization of its derivatives like chitooligosaccharides and chitosan-based nanomaterials, films, fibers, and composites. The obtained bioactive polymers reported various therapeutic applications as antioxidants, antibiotics, anticancer agents, dietary fibers, etc. [120,121]. At present, several commercially available chitin and chitosan-based nutraceutical products from companies (such as Kitto Life, Natural Balance, Universal Nutrition, Primex, Chitopower, etc.) are available, which focus on therapeutic properties in addition to weight loss, immune enhancer, cholesterol controlling, antioxidant, osteoarthritis, dietary fiber, dietary supplements, etc. [120,122].

### 6.7. Chondroitin, Glucosamine, and Hyaluronic Acid

Chondroitin sulfate, glucosamine sulfate, and hyaluronic acid are glycosaminoglycans synthesized by chondrocytes and synoviocytes, and are the basic components of the extracellular matrix and synovial fluid [123]. Chondroitin sulfate is an important structural component of cartilage and provides much of its resistance to compression [124]. Glucosamine is a crystalline compound which occurs widely in connective tissue, especially as a component of chitin. As such, research indicates that utilizing chondroitin sulfate and glucosamine sulfate in a combined formula is very effective for treatment of osteoarthritis (OA) and relieving arthritis pain. Both support cartilage matrix and can function conjointly in benefitting OA treatment and have become a widely used dietary supplement [71]. Glucosamine inhibits some of the enzymes responsible for cartilage resorption (phospholipase A2 and collagenase). With these characteristics, glucosamine sulfate supports the regulation of out-of-balance metabolism of cartilage generation and resorption found in OA. Furthermore, glucosamine sulfate has anti-inflammatory characteristics as well. [72]. 

Additionally, hyaluronic acid derived from fish or fish products has a wide range of applications and is involved in many biochemical processes, including cell signaling, wound reparation, tissue regeneration, and morphogenesis [125]. Its physico-chemical properties such as biodegradability, biocompatibility, non-toxicity, and non-immunogenicity serve as excellent tools in biomedical applications such as osteoarthritis surgery, ocular surgery, plastic surgery, tissue engineering, and drug delivery. It plays a key role in cushioning and lubricating the body and is abundant in the eyes, joints, and heart valves [123,126]. Nutraceutical application of glucosamine sulfate (20 mg/kg body weight/day), chondroitin sulfate (1200 mg/d), and hyaluronic acid (50–100 mg/d) can be helpful in providing anti-inflammatory and redox balance or antioxidant, anabolic properties, high anticatabolic with low, moderate and high activity, respectively [123].

### 6.8. Gelatin and Collagen

Gelatin, an important colorless and tasteless biopolymer obtained from the heating of collagen above the transition temperature [74]. Fish gelatin extracted from warm-water fish possesses similar characteristics to porcine gelatin and may thus can be considered as an alternative to mammalian gelatin for use in pharmaceutical products. Fish gelatin with low melting points could be used in the microencapsulation of vitamins and other pharmaceutical applications [73]. Moreover, collagen is the main structural protein in the animal kingdom, and it is estimated that in fish processing the residue after filleting may be 75% of the total weight, of which a large part consists of skin and bones with high quantities of this protein. Such large amounts of by-products could be used for a wide range of applications. Collagen is characterized by its high content of glycine, proline, and hydroxyproline, denatured in the presence of dilute acid standards and converted to soluble protein such as gelatin, when dissolved in heated solutions. Derived from collagen, gelatin has many applications in food, pharmaceutical, photographic, and other products [74]. In recent years, the rising interest in halal products for Muslim populations has become one of the main reasons for exploring different types of collagen and gelatin from different animal sources [73]. Similarly, collagen plays an important role in the formation of tissues and organs and is involved in various functional expressions of cells. Their applications include treatment of pain associated with osteoarthritis, hypertension, use in tissue engineering, implants in human, and inhibition of angiogenic diseases. It is also used as dermal filler, as hemostat, for drug delivery, skin substitutes, expandable intra-arterial stents, and as a cell attachment substrate [76].

### 6.9. Squalene

Squalene is considered as an important natural molecule with isoprenoid hydrocarbon and is synthesized in plants, animals, and bacteria as a precursor for the synthesis of secondary metabolites like vitamins, sterols, or hormones. Moreover, the best source of squalene is the liver of the deep-sea shark. On average, almost 2300–8400 mg/100 g of squalene is usually extracted from shark liver oil. Squalene has been found to have broad applications in the food industry and cosmetics as well as in prevention and treatment of human diseases (immunity booster; anticancer effects against ovarian, breast, lung, and colon cancer; skin disorders; reduces skin damage by UV radiation; increases stamina, LDL levels, and cholesterol in the blood; prevents the suffering of cardiovascular diseases; antioxidant, antibacterial and antifungal) [78,127,128]. It also improves digestive health due to the increased production of bile acids and helps in normalization of constipation and diarrhea, which makes it effective for the treatment of gastritis. Balancing of hormone levels through its involvement in the production of steroid hormones, resulted in increased sexual vitality and improvement in pre-menstrual syndrome, menopausal problems, and even fertility [77]. Furthermore, it also minimizes the side-effects of drugs through its detoxifying action [129]. Concurrently, squalene is also recommended to add to the human diet due to its various nutraceutical properties that do not cause health risks [127]. Squalene supplements as soft gel capsule from Best Nutrition Products (Hayward, CA, USA) are commercially available for cardiovascular, heart health and joint problems.

## 7. Challenges and Complications

Potential health benefits of fish and its therapeutic effect have been well documented. However, considerable ambiguity regarding the toxicological as well as environmental harms cannot be ignored. Among the hundreds of fish species available for consumption, a wide variety of fish with different levels of contaminants can be found. It has been noticed that a significant level of different toxicants such as methyl mercury, polychlorinated biphenyls, dioxins, organochlorine pesticides, aldrin, chlordane, dieldrin, mirex, toxaphene, and other environmental contaminants present in fish cause a decline in fish consumption [130,131]. The mercury concentrates present in fish are predominantly present in the form of methyl mercury which is considered as neurotoxic; this could affect neurodevelopment in children and chronic exposure of mercury even at low concentrations can cause cardiovascular, reproductive illnesses, developmental toxicity, neurotoxicity, nephrotoxicity, immunotoxicity, and carcinogenicity [132,133]. Other than fish contaminants, there are several factors which pose challenges for consumption of fish such as social values, religion, cultural influence, cost, and accessibility as well as education. Social and cultural influences lead to differences in habitual consumption of certain foods and in certain cases can lead to restrictions such as exclusion of fish and other meat product in the diet. Sometimes, social determinants such as culture, family, peers, and meal patterns also affect eating habits [134].

Due to the potential health benefits of fish, the Dietary Guidelines for Americans, 2015–2020 (DGA) recommend that people consume 8 oz of seafood per week (less for young children) and woman who are pregnant or breast feeding should consume between 8 and 12 oz of a variety of seafood per week, from choices that are lower in mercury [135] especially marine-derived “oily” fish such as salmon, mackerel, sardines, pompano, anchovies, swordfish, trout, and tuna, to provide an average daily consumption of 250 mg of EPA/DHA per day [136]. The DGA also recommend consumption of a variety of types of seafood to reduce the amount of methyl mercury consumed from any one type. Five of the top 10 consumed seafood are low in mercury: shrimp, light tuna, salmon, pollock, and catfish [137]. Therefore, when choosing fish for consumption, one must be aware of the properties of the fish as well as risk factors. Considering that there are many fish species, it is recommended to use the advisories to learn more about the fish that one consumes, to choose fish with higher levels of omega-3 PUFAs, to avoid or minimize choosing fish with potentially higher levels of contaminants, and to consume a variety of fish.

## 8. Future Perspectives and Conclusions

What we eat influences our health. Historically fish has been known as a healthy food. Recently researchers have opened the possibilities of extracting rare and useful nutraceuticals from fish. The large amount of fish bioactive components has been explored for their therapeutic efficacy. However, based upon the searched data in PubMed for the last 20 years, there is very little information available for fish-based nutraceuticals when compared to published articles on nutraceuticals and functional food in general. Therefore, this prevailing data suggest that even growing concern and awareness about the nutritional as well as therapeutic application of fish-based nutraceuticals have not been properly explored for its potential. Fisheries and aquaculture must address many of these difficult challenges. Especially with rapidly expanding aquaculture production around the world, there is a large potential of further and rapid increases in fish supply, an important source of animal protein for human consumption. Thus, even in small quantities, provision of fish can be effective in addressing food and nutritional security among the poor and vulnerable populations around the globe as well as bioactive components present in fish could prove important for human diet as they present numerous health effects. This can be utilized as a holistic approach with a concept of converting a fish-based nutraceutical to a medicinal food with not only the intention of satisfying global hunger, but also to provide bioactive ingredients and essential macro and micronutrients to the body that can aid to decrease nutrition-related diseases and ensure physical and mental well-being.

## Figures and Tables

**Table 1:** List of nutraceutical/bioactive components of fish, fish wastes, and fish by-products with their health applications.

Nutraceutical/Bioactive Components	Fish Species Rich in Specific Bioactive Components	Therapeutic Efficacy and Clinical Implications	References
Fatty Acids			
Omega 3	Mackerel, Spiny dogfish, Black halibut, Salmon, Sardines	Anti-inflammatory, cardio-protective effects, visual and neurodevelopment, different cancers (breast, colorectal, prostate, etc.), asthma, inflammatory bowel disease, rheumatoid arthritis and osteoporosis, improve insulin sensitivity	[9,42,43,44,45]
Omega 6	Arctic Char, Sardine, Fried Calamari, Anchovies	Reduce risk of cardiovascular problems, ameliorate diseases such as arthritis and hypertension, increases vascular adhesion molecule-1 expression, oxidation, platelet aggregation, vasoconstriction, eicosanoid synthesis	[9,43,45]
Amino Acids			
Arginine	*Oncorhynchus mykiss*	Required for the detoxification of ammonia, nutritionally essential for spermatogenesis, embryonic survival, fetal and neonatal growth, as well as maintenance of vascular tone and hemodynamics	[46]
Histidine	*Rastrelliger kanagurta, Catla catla*	Precursor for several hormones (thyrotropin-release hormone), critical metabolite for renal functions, neurotransmission, gastric secretion and immune system, antioxidant and anti-inflammatory properties, important for the regulation and metabolism of trace elements as well as precursor of histamine	[47,48]
Isoleucine	*Oncorhynchusmykiss, Labeo rohita*	Help in muscle formation and proper growth	[49]
Lysine	*Stolephorus waitei, Rastrelliger kanagurta, S. commersonii*	Needed for optimal growth and to act as an immunomodulator, prevention and treatment of cold sores	[49]
Methionine	*Stolephorus waitei, Tor putitora*	Used at multiple levels in cellular metabolism, as a protein constituent, in the initiation of mRNA translation, and as a regulatory molecule in the form of S-adenosylmethionine	[50]
Phenylalanine	*Cirrhinus mrigala, Catla catla*	Precursor for tyrosine	[51]
Threonine	*Thunnus albacores, Nemipterus japonicus*	Plays a critical role in the maintenance of intestinal mucosal integrity and barrier function	[52]
Tyrosine	*Oncorhynchus mykiss, Tor putitora*	Precursor of dopamine and norepinephrine	[53]
Valine	*Nemipterus japonicas, Cirrhinus mrigala*	Protein synthesis, glucose homeostasis, anti-obesity, and nutrient-sensitive signaling pathways	[54]
Glutamine	*Cirrhinus mrigala, Catla catla, Labeo rohita*	Act as substrate for nucleotide synthesis (purines, pyrimidines, and amino sugars), nicotinamide adenine dinucleotide phosphate (NADPH), antioxidants, and many other biosynthetic pathways involved in the maintenance of cellular integrity and function	[55]
Glycine	*Cirrhinus mrigala, Catla catla, Labeo rohita*	Help in regulation of gene expression, protein configuration and activity, and several biological functions, such as glutathione synthesis. Low plasma glycine concentrations have been consistently reported in association with obesity and type 2 diabetes	[56,57]
Proline	*Oncorhynchus mykiss, Tor putitora*	Important role in differentiation of cells as well as fetus and associated with extra-embryonic membrane and development	[58]
Alanine	*Polypedates maculates*	Helps in biosynthesis of proteins, serves as an important carbon source for hepatic gluconeogenesis	[59]
Aspartic acid	*Labeo niloticus*	Treatment for chronic fatigue due to the role it plays in generating cellular energy	[59]
Glutamic acid	*Labeo niloticus*	Surfactants, buffer, chelating agents, flavor enhancer, agriculture, acts as fuel, immune function	[59,60]
Leucine	*Lethrinus harak*	Leucine promotes energy metabolism (glucose uptake, mitochondrial biogenesis, and fatty acid oxidation) to provide energy for protein synthesis, while inhibiting protein degradation	[59,61]
Serine	*Mugil cephalus*	Cellular homeostasis, proliferation, and differentiation	[59,62]
Vitamins			
Vitamin A	*Amblypharyngodon mola*	Growth promoter, helps in poor eyesight, helps in bone growth	[3,6]
Vitamin D	*Amblypharyngodon mola, Sardinella longiceps*	Rickets, osteomalacia, improve bone density	[3,13]
Vitamin B Complex	Black sea fish, Shellfish	Responsible for converting food to energy in the cells of the body and helps with the function of nerve tissue	[6,63]
Minerals			
Iron	*Sperata seenghala, Rita rita*	Help in synthesis of hemoglobin in red blood cells	[13]
Zinc	*Sperata seenghala, Rita rita*	Important role in growth and development as well in the proper functioning of the immune system and for healthy skin. Helps in cell division, cell growth, wound healing, and breakdown of carbohydrates. Essential for senses of smell and taste	[6]
Calcium	*Xenentodon cancila, Gudusia chapra*	Essential for strong bones (formation and mineralization) and for the normal functioning of muscles and the nervous system	[6]
Iodine	-	Important for hormones that regulate body metabolism, and in children it is required for growth and normal mental development	[6]
Carotenoids			
Astaxanthine, Beta carotene, Zeaxanthin and lutein	Freshwater fish, red fishes, and other fishes	Antioxidant, cancer, neurological disorder, cardiovascular, anti-atherogenic, ophthalmology, psoriasis, preservative, cosmetics	[64,65]
*Other Bioactive Components*			
Bioactive Peptides	Fish protein, fish by-products, and muscle	Antihypertensive, antioxidant, antimicrobials, and anti-proliferative effects	[66,67]
Chitin and Chitosan	Fish waste product (fish scale)	Wound healing accelerator, reduces blood cholesterol levels, anti-ulcer agent, anti-ageing, cosmetics, ophthalmology	[68,69,70]
Chondroitin	Fish waste product	Osteoarthritis, dietary supplement	[71]
Glucosamine	Fish waste product	Anti-inflammatory, osteoarthritis, dietary supplement	[71,72]
Gelatin	Fish waste product	Pharmaceutical industries, food industries, microencapsulation of vitamin, stabilizer in dairy products, cosmetics	[73,74,75,76]
Collagen	Fish waste product	In osteoarthritis, hypertension, tissue engineering, antioxidant, anti-hypertensive, and anti-skin ageing	[9,76]
Squalene	*Scardinius erthrophthalmus, Tinca tinca*	Cardio-protective, antioxidant, anti-bacterial, antifungal, and anticancer	[77,78,79]
